# Bioinformatics Analysis of the Molecular Mechanism of Late-Stage Heterotopic Ossification

**DOI:** 10.1155/2020/5097823

**Published:** 2020-04-11

**Authors:** Qiang Zhang, Yan Zhang, Meijun Yan, Kai Zhu, Dong Zhou, Jun Tan

**Affiliations:** ^1^Department of Orthopaedic Surgery, Shanghai East Hospital, Tongji University School of Medicine, 150 Jimo Rd, Pudong New District, Shanghai 200120, China; ^2^Department of Orthopaedic Surgery, The Affiliated Changzhou No. 2 People's Hospital with Nanjing Medical University, Changzhou, Jiangsu, China; ^3^Department of Orthopedics, Pinghu Second People's Hospital, Pinghu 314200, China

## Abstract

**Background:**

Heterotopic ossification (HO) is a common disease happened in soft tissues after injury. The present study utilized the bioinformatics method to analyze the HO samples in a mouse burn/tenotomy-induced HO model to identify the possible key points and treatment targets.

**Methods:**

The transcriptome profiles of GSE126118 were obtained from the Gene Expression Omnibus (GEO) database. The study was based on a mouse burn/tenotomy-induced HO model, and 2 tenotomy samples and 3 uninjured contralateral hindlimb tendon samples were collected at 3 weeks after injury for further analysis. The transcripts per million approach was performed for background correction and normalization; then, the differentially expressed genes (DEGs) were detected using the limma R package with the settings *p* < 0.01 and ∣log2FC∣ > 2.0. The Gene Ontology (GO), Kyoto Encyclopedia of Genes and Genomes (KEGG) pathway enrichment, and the protein-protein interaction (PPI) network analysis were performed with the detected DEGs.

**Results:**

A total of 74 DEGs were upregulated, and 159 DEGs were downregulated between the tenotomy and uninjured tendon group. Pathway and process enrichment analyses demonstrated that the upregulated DEGs were mainly associated with terms related to ECM remodeling, ossification, angiogenesis, inflammation, etc., and the downregulated DEGs were mainly associated with oxidative phosphorylation, metabolic process, etc.

**Conclusion:**

The results of GO, KEGG, and PPI network analyses suggested that the ECM remodeling, ossification, angiogenesis, and inflammation processes were markedly upregulated in the tenotomy site. And the oxidative phosphorylation and metabolic processes were markedly downregulated. These findings provide valuable clues for highlighting the characteristics of late-stage HO and investigating possible treatments.

## 1. Introduction

Heterotopic ossification (HO) is a common disease happened in soft tissues after injury [1]. Both acquired and hereditary HO cases have been widely discussed, with no effective treatment developed yet. Unlike the low incidence of hereditary HO, a much higher incidence is observed with the acquired HO due to the injury and failed tissue repair. The acquired HO mostly happens after local or systemic injury/inflammatory, for example, orthopedic surgery, burns, brain/spinal cord injury, injury, and even immunity-related diseases. Nearly all the acquired HO developed via endochondral ossification, a biological process usually seen in both bone development and fracture healing. The heterotopic endochondral ossification process triggers with injury/inflammatory, following mesenchymal stromal cell (MSC) recruitment, chondrogenic differentiation, and finally ossification formation [2]. For preventing HO, the inhibition of the chondrogenic differentiation process is most important considering the balance between promoting regeneration and suppression ossification; however, for treating the late-stage HO, the critical points may be the inhibition of osteogenesis and promoting ossification absorption. Unluckily, neither preventing nor treating HO methods has been achieved effectively yet. In late-stage HO, various biological processes are involved, including inflammatory, chondrogenesis, osteogenesis, angiogenesis, mineralization, and bone absorbing. The strategies for treating late-stage HO have been widely investigated with various biological processes. For example, low-dose radiation has been widely used in treating HO with the ability to kill the chondrogenic and osteogenic cells [3, 4]. The anti-inflammatory agent cyclo-oxygenase-2 inhibitors was able to inhibit osteogenic differentiation of MSCs besides the ability to inhibit inflammatory that triggers HO [5, 6]. Similarly, the BMP pathway inhibitors and RAR*γ* agonists were also able to reduce chondrogenesis and thus heterotopic endochondral ossification [7–11]. But unfortunately, none of the treatments is useful enough now.

The present study utilized the bioinformatics method to analyze the HO samples in a mouse burn/tenotomy-induced HO model to identify the possible key points and treatment targets. Specifically, differentially expressed genes (DEGs) of the HO samples and the uninjured contralateral tendon samples were evaluated by pathway and functional enrichment analysis. A protein-protein interaction (PPI) network was then constructed using these DEGs. These analyses revealed several molecular mechanisms that may contribute to late-stage heterotopic ossification.

## 2. Method

### 2.1. Data Source

The transcriptome profiles of GSE126118 were obtained from the National Centre of Biotechnology Information (NCBI) Gene Expression Omnibus database (GEO, https://www.ncbi.nlm.nih.gov/geo/). GSE126118, which comprises a total of 7 chips, including 2 tenotomy samples, 3 uninjured contralateral hindlimb tendon samples, and 2 normal tendon samples, was based on the platform of the GPL13112 Illumina HiSeq 2000 (Mus musculus). The study was based on a mouse burn/tenotomy-induced HO model, briefly, a partial-thickness scald burn injury together with a transection tenotomy at the midpoint of the Achilles tendon. For each group, all the mice belong to the same kind and batch, and all the surgery procedures were performed by the same person at the same time. The tendon samples were collected, and total RNA was isolated at 3 weeks after injury.

### 2.2. Data Preprocessing and Differential Expression Analysis

The transcripts per million (TPM) approach was performed for background correction and normalization of the raw data of the dataset; then, the differentially expressed genes (DEGs) were detected using the limma R package. The DGEs were defined with the settings *p* < 0.01 and ∣log2FC∣ > 2.0 based on Benjamini and Hochberg (BH) procedure. And the Ensembl transcript IDs were then converted into gene symbols, and if different probes were annotated to the same gene, the average value was served as the gene's expression level. The heat map was also drawn by the online tool Morpheus (https://software.broadinstitute.org/morpheus/).

### 2.3. Pathway and Functional Enrichment Analysis

The Metascape (http://metascape.org/gp/index.html) was used to perform the Gene Ontology (GO) and Kyoto Encyclopedia of Genes and Genomes (KEGG) pathway enrichment analysis. The enriched pathways were obtained to analyze the common DEGs at the functional level with the setting *p* < 0.05.

### 2.4. PPI Network Construction

The protein-protein interaction (PPI) network was analyzed and visualized by STRING (https://string-db.org). The network data from the STRING database displayed a combination of data, including text mining, neighborhood, coexpression, co-occurrence, gene fusion, and experiments. The score of minimum required interaction was medium confidence (0.400).

## 3. Results

### 3.1. Data Preprocessing and DEG Screening

**Figure 1:**
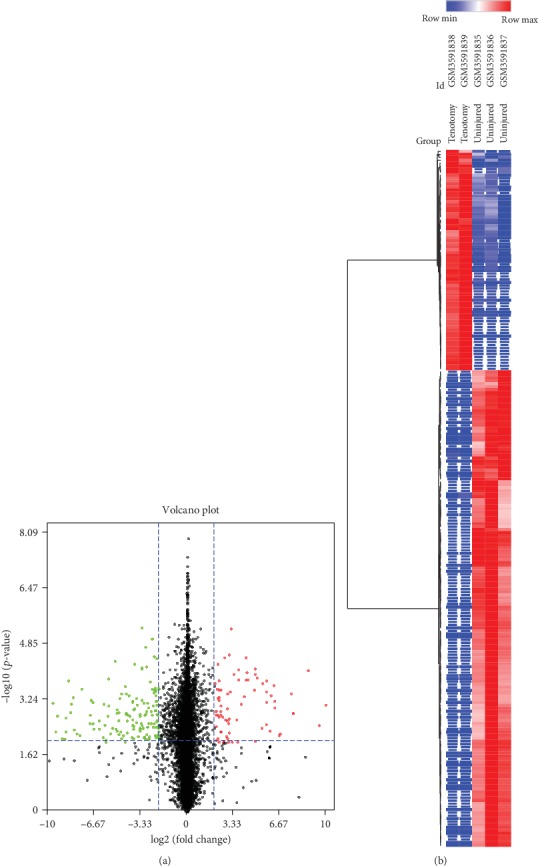
(a) Volcano plots of the DEGs between the tenotomy group and the uninjured contralateral tendon group. (b) Finally, heat map of the DEGs between the tenotomy and uninjured tendon groups. Horizontal axis represents each sample, and the vertical axis represents each gene. Blue and red colors represent low and high expression values, respectively.

The expression data of the dataset GSE126118 was normalized with the TPM approach. The DEGs between the tenotomy group and the uninjured contralateral tendon group were then analyzed. A total of 74 DEGs were upregulated, and 159 DEGs were downregulated with the cut-off setting of *p*-value < 0.01 and ∣log2FC∣ > 2.0. Volcano plots were used to visualize the identified DEGs (Figure 1(a)). Finally, heat maps of gene expression values were constructed with color patterns indicating the variability in gene expression between the tenotomy and uninjured tendon groups (Figure 1(b)).

### 3.2. Pathway and Functional Enrichment Analysis

**Figure 2:**
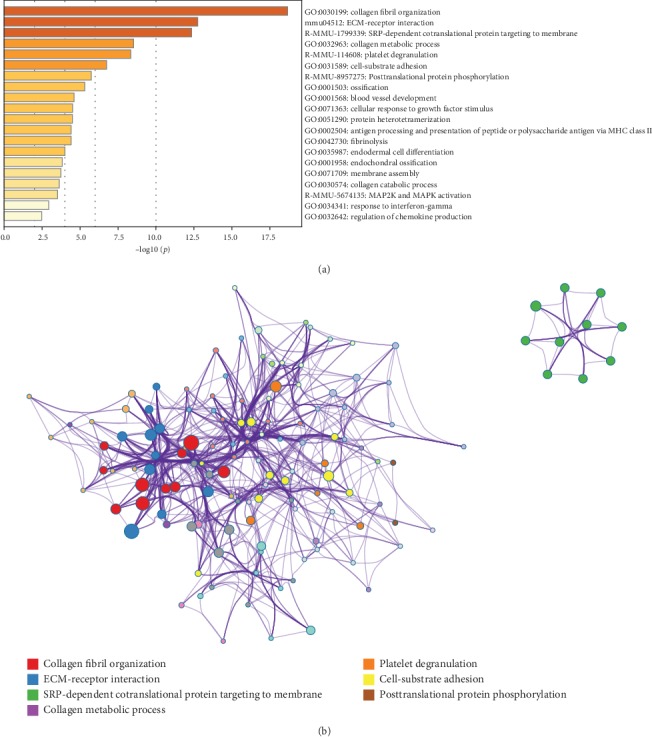
Pathway and process enrichment analysis of upregulated DEGs. (a) Heat map of enriched terms related to the inputted list of genes, colored according to their *p*-values. (b) The network of enriched terms. Each term is represented by a circle node, where its size is proportional to the number of input genes fall into that term, and its color represent its cluster identity (i.e., nodes of the same color belong to the same cluster). Terms with a similarity score > 0.3 are linked by an edge (the thickness of the edge represents the similarity score).

**Figure 3:**
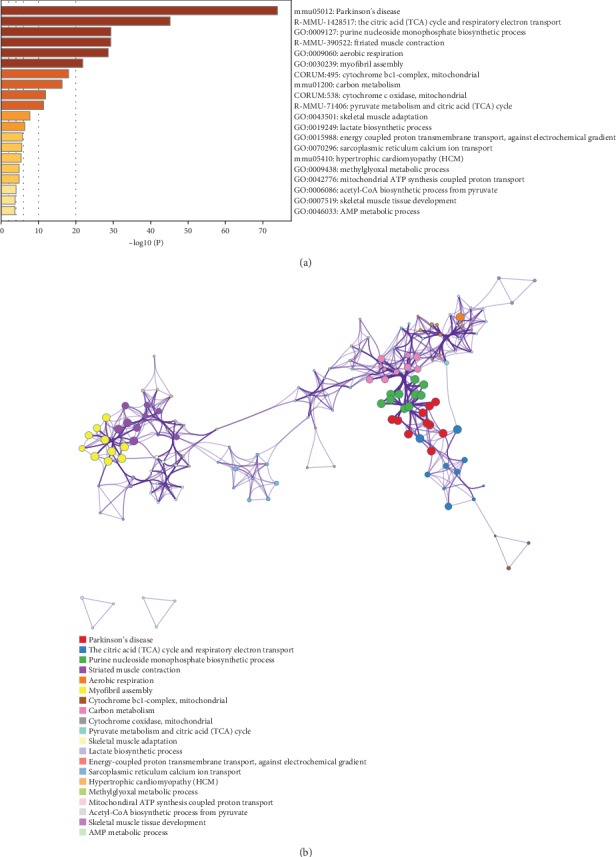
Pathway and process enrichment analysis of downregulated DEGs. (a) Heat map of enriched terms related to the inputted list of genes, colored according to their *p*-values. (b) The network of enriched terms. Each term is represented by a circle node, where its size is proportional to the number of input genes fall into that term, and its color represents its cluster identity (i.e., nodes of the same color belong to the same cluster). Terms with a similarity score > 0.3 are linked by an edge (the thickness of the edge represents the similarity score).

Pathway and process enrichment analyses were carried out with the following ontology sources in Metascape: KEGG pathways and GO biological processes. All genes in the genome were used as the enrichment background. Terms with *p* < 0.01, minimum count = 3, and enrichment factor (ratio between observed count and count expected by chance) > 1.5 were collected and grouped into clusters based on membership similarities. More specifically, *p*-values were calculated based on accumulative hypergeometric distribution, *q*-values were calculated using the Benjamini–Hochberg procedure to account for multiple testing (Hochberg and Benjamini, 1990), kappa scores were used as the similarity metric when performing hierarchical clustering of the enriched terms, and subtrees with similarity > 0.3 were considered a cluster. Regarding the hierarchical clustering of the enriched pathway and process terms, the most statistically significant term within a cluster was selected for cluster annotation. We found that the upregulated DEGs were mainly associated with terms related to ECM remodeling, ossification, angiogenesis, inflammation, etc. (Figures 2(a) and 2(b)), and the downregulated DEGs were mainly associated with oxidative phosphorylation, metabolic process, etc. (Figures 3(a) and 3(b)).

### 3.3. PPI Network Analysis

**Figure 4:**
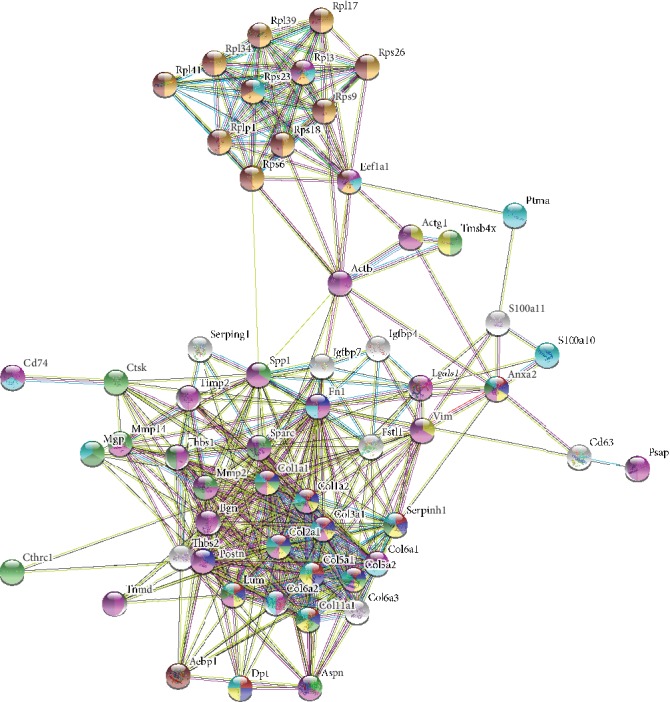
Analysis of protein-protein interaction (PPI) by online bioinformatics. The PPI of DEPs analyzed against the STRING database for association networks. Known interactions are edges of pink (experimentally determined) and deep sky blue (database obtained). Predicted interactions are edges of green (gene neighborhood), blue (gene co-occurrence), and red (gene fusions). Edges of yellowish green are text mining. Edges of black color mean coexpression. Edges of light purple mean protein homology.

To explore PPI networks among the identified proteins, the identified DEGs were analyzed using the STRING software. The DEGs that did not connect to any type of network were excluded (STRING interaction score < 0.4) (Figure 4).

## 4. Discussion

The present study aimed to explore underlying mechanisms related to the late-stage heterotopic ossification. GSE126118 public transcriptome data from the Gene Expression Omnibus database were used for analysis. First, raw data were normalized, and differentially expressed genes (DEGs) were identified. Next, the Kyoto Encyclopedia of Genes and Genomes (KEGG) and Gene Ontology (GO) analyses were implemented to evaluate pathways and DEGs. A protein-protein interaction (PPI) network was then constructed. A total of 74 DEGs were upregulated, and 159 DEGs were downregulated between the injured and the uninjured contralateral tendon. The results of GO, KEGG, and PPI network analyses suggested that the ECM remodeling, ossification, angiogenesis, and inflammation processes were markedly upregulated in the tenotomy site. These findings provide valuable clues for highlighting the characteristics of late-stage HO and investigating possible treatments.

The ECM remodeling is important in HO pathologies, no matter for tissue regeneration or chondrogenic/osteogenic differentiation. The tendon tissue consists mainly of extracellular matrix [12], which accounts for 70–80% of the dry weight, including collagen, elastin, and proteoglycans. The type I collagen accounts for 97–98% of the total collagen [13], together with a small number of other types of collagens like type II, III, IX, V, and X collagen. For cartilage or fibrocartilage tissue, ECM is also the major content, which is made up of glycosaminoglycans, proteoglycans, collagen fibers, and sometimes, elastin. Unlike tendon, the type II collagen is the majority collagen type of cartilage, together with aggrecan as the main proteoglycan. For bone tissues, it is more complicated. The bone tissue is made up of different types of bone cells. Osteoblasts and osteocytes are involved in the formation and mineralization of bone; osteoclasts are involved in the resorption of bone tissue. Bone consists of a flexible matrix (about 30%) and bound minerals (about 70%), the bone matrix is 90 to 95% composed of elastic collagen fibers, also known as ossein, and the remainder is ground substance. The process of HO involves a complicated transfer of the ECM from tendon to bone, and the process is mainly triggered and maintained by the transfer of the cells from tenocytes to chondrocytes and finally to osteoblast, and most importantly, the differentiation into chondrocytes contributes mainly to the ECM remodeling.

The origin of chondrocytes remains uncertain. Lots of hypotheses have been raised, and the erroneous differentiation of stem cells is believed responsible for it. MSCs were recruited to the injury site, influenced by the microenvironmental niche, and finally differentiated into the chondrocytes [14, 15]. Till now, no consequence has been got regarding the origin type of progenitors, including tissue-resident progenitor cells, mesenchyme stem cells recruited from the circulation, vascular endothelial cells, and endoneurium cells [14–18]. For the tendon ossification, the tissue-resident progenitor cells, namely, the tendon derived stem cell, were considered most important. During the late stages of HO, the major biological process is endochondral ossification, an essential process during fetal development and fracture healing. Typically, the cartilage model has already formed in the late stage, and it grows in length by continuous cell division of chondrocytes. Then, the first site of ossification occurs with the hypertrophy of the chondrocytes and differentiation of osteoblasts. The inhibition of chondrocytes proliferation and osteogenic differentiation seems to be useful for treating late-stage HO. The low-dose radiation can kill or induce senescence to the target cells, thus, reducing the proliferation and differentiation. And the implication of RAR*γ* agonists was also able to reduce chondrogenesis and thus heterotopic endochondral ossification [7–11]. Similarly, considering the importance of HedgeHog signaling in osteogenesis, the use of HH signaling inhibitors has also been widely researched [19]. Drugs such as arsenic trioxide (ATO) and GANT58 have been found to reduce HO in the progressive osseous heteroplasia models [19–21].

Angiogenesis is also important in heterotopic ossification pathologies, including significant vascular modeling and remodeling. For early-stage HO, angiogenesis is obvious with the highest number of capillaries. When the cartilaginous area forms, the number of vessels drops significantly with a small number of vessels locating around the avascular cartilage callus. However, for late-stage HO, a high number of vessels with variably enlarged size can be observed; when the ossification matures, there are fewer vessels with the largest size. The angiogenesis is quite important in HO, with the ability of bringing immune signaling, growth factors, nutrition, etc. Inhibition of angiogenesis with either a small molecule (TNP-470) or a targeted biological (vascular endothelial growth factor receptor type 2 [VEGFR2] blocking antibody) prevented ectopic bone formation by 83% and 77%, respectively, either inhibiting chondrogenesis or proliferate prior to hypertrophy, as well as osteoclast recruitment and resorption were almost completely inhibited [22].

The inflammatory is also important in late-stage HO, though much weaker than the early stage. The osteoclasts, one of the immune cells, are extremely important in ectopic bone remodeling [23]. Increased bone breakdown by osteoclasts leads to increased bone formation in HO. The immune system is known to have a wide range of effects on osteoclastogenesis via a wide variety of cells, which in turn affects HO formation. The anti-inflammatory agents are always useful in preventing and reducing bone formation for all stages of HO. And the cyclo-oxygenase-2 inhibitors were able to inhibit osteogenic differentiation of MSCs besides the ability to inhibit inflammatory that triggers HO [5, 6].

The enrichment of the downregulated gene was mainly focused on the oxidative phosphorylation and metabolic processes, which may due to the different cell types between the tissues. No research exploring the relationship between oxidative phosphorylation/metabolic processes and HO has been conducted yet; further studies should be made towards it.

The present study utilized a bioinformatic method to identify the possible key points in late-stage heterotopic ossification. However, several limitations exist with the study, one limitation is the small number of sample size and datasets included, which limits the accuracy of the conclusions. Another limitation is the present study failed to identify an exact protein/pathway that contributes mainly to the HO. Further studies are required to determine the molecular mechanisms by which these biological events participate in HO.

## 5. Conclusion

The present study utilized the bioinformatics method to analyze the HO samples in a mouse burn/tenotomy-induced HO model to identify the possible key points and treatment targets. A total of 74 DEGs were upregulated, and 159 DEGs were downregulated between the injured and the uninjured contralateral tendon. The results of GO, KEGG, and PPI network analyses suggested that the ECM remodeling, ossification, angiogenesis, and inflammation processes were markedly upregulated in the tenotomy site. And the oxidative phosphorylation and metabolic processes were markedly downregulated. These findings provide valuable clues for highlighting the characteristics of late-stage HO and investigating possible treatments.

## Data Availability

The original data used to support the findings of this study are available at GEO dataset (GSE126118).
